# Uterine Perforation by Levonorgestrel-Releasing Intrauterine Device: A Case Report

**DOI:** 10.7759/cureus.31398

**Published:** 2022-11-12

**Authors:** Jose D Roman

**Affiliations:** 1 Gynaecology Department, Braemar Hospital, Hamilton, NZL

**Keywords:** diagnosis of uterine perforation by iud, management of uterine perforation by iud, complications of iud, uterine perforation by intrauterine device, levonorgestrel-releasing intrauterine device

## Abstract

The intrauterine device (IUD) is the most popular method of reversible contraception in women of reproductive age nowadays, and its use is being reported by nearly 160 million women. It is considered safe and effective, but intrauterine devices are associated with rare complications such as uterine perforation. We report a case of uterine perforation by a levonorgestrel-releasing intrauterine device (LNG-IUD), Mirena (Bayer, Auckland, New Zealand) levonorgestrel 52 mg, to create awareness of the diagnosis and the laparoscopic management of these rare cases.

## Introduction

The first report on the successful prevention of human conception with an intrauterine device (IUD) was made by Dr. Richard Richter of Poland in 1909 [1]. He made a flexible ring of silkworm gut and cut off the two ends at the level of the cervix to make the examination and removal easier later on [2]. Nowadays, 17% (159 million) of women of reproductive age (15-49 years) use an IUD as a contraceptive. It has become the second most common form of contraception after sterilization [3].

The safety and effectiveness of modern intrauterine devices have been improved, and they are considered nowadays a low-risk method of birth control. However, some rare complications such as uterine perforation may have dangerous consequences. Physician awareness is crucial in how to diagnose and manage a patient with such a complication.

## Case presentation

A 32-year-old woman was referred to our clinic because of an atypical squamous cell smear result and a positive high-risk human papillomavirus (HPV) test. Her previous cervical smears were reported as normal, and she was fully vaccinated with the HPV Gardasil vaccine.

Her periods were regular and recently lighter than usual after she had a levonorgestrel-releasing intrauterine device (LNG-IUD), Mirena (Bayer, Auckland, New Zealand) levonorgestrel 52 mg, inserted the previous year to treat her menorrhagia. She had two children, the youngest one was two years old and born by an uncomplicated cesarean section.

**Figure 1:**
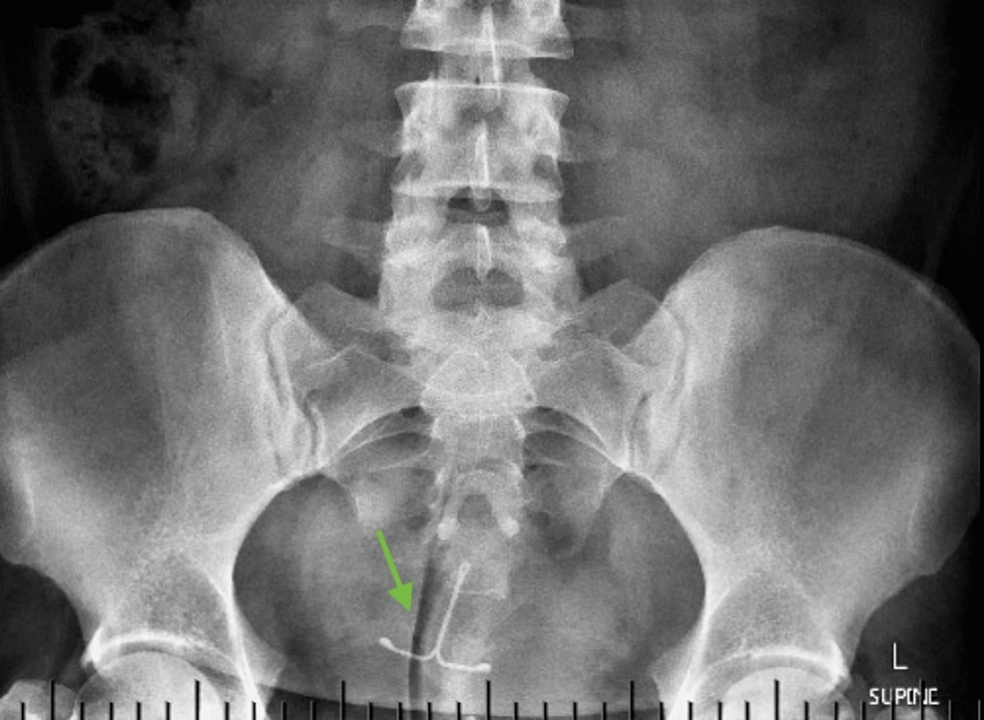
Erect X-ray of the patient revealing the LNG-IUD “inside” the pelvic cavity in an “upside down” position in the vicinity of the uterus. LNG-IUD: levonorgestrel-releasing intrauterine device

The colposcopic biopsy revealed cervical intraepithelial neoplasia (CIN) 3, and as the LNG-IUD threads were not visible during the examination, a vaginal pelvic ultrasound was requested that revealed a retroverted normal-sized uterus with an endometrial thickness of 4 mm and an empty uterine cavity. After a serum beta-human chorionic gonadotropin (BHCG) was reported as negative, an abdominopelvic anteroposterior and sagittal X-ray was requested that revealed a satisfactory bowel gas pattern, and the above IUD was showed to be in the central pelvis in the proximity of the uterus (Figure 1).

At the preoperative consultation, she was booked for hysteroscopy and operative laparoscopy +/- laparotomy. Full blood count and renal function tests were requested, and full bowel preparation was arranged with sodium picosulfate and magnesium oxide laxative (PicoprepR) prior to surgery.

**Figure 2:**
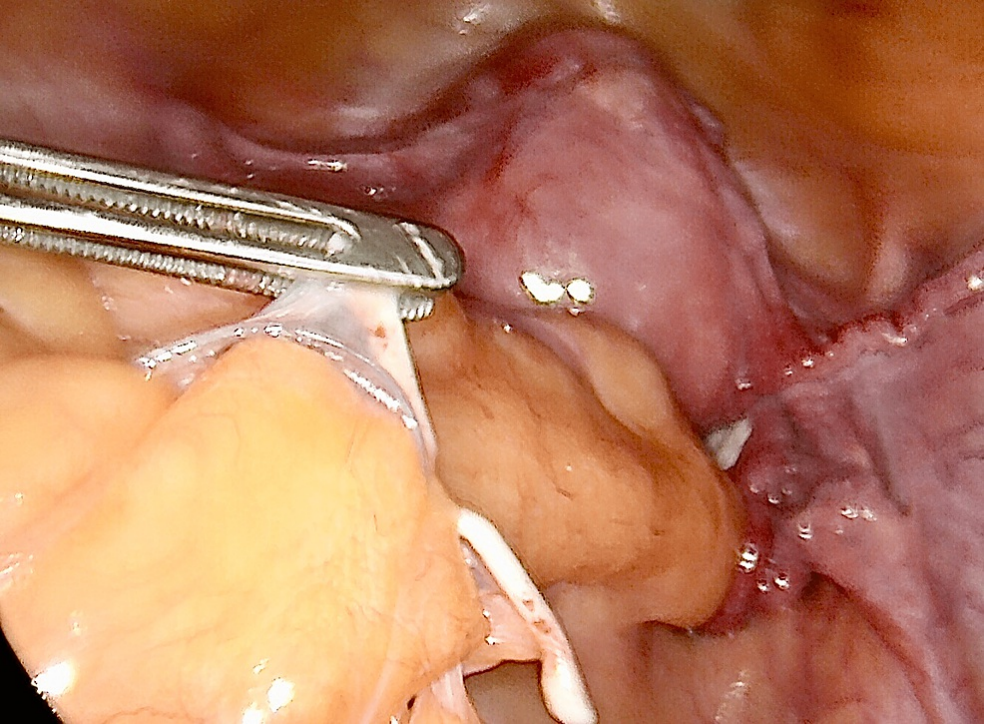
A LNG-IUD densely attached to the fat cover of the sigmoid colon is located at the level of the pelvic brim. The retroverted, retroflexed uterus can be seen further down. LNG-IUD: levonorgestrel-releasing intrauterine device

**Figure 3:**
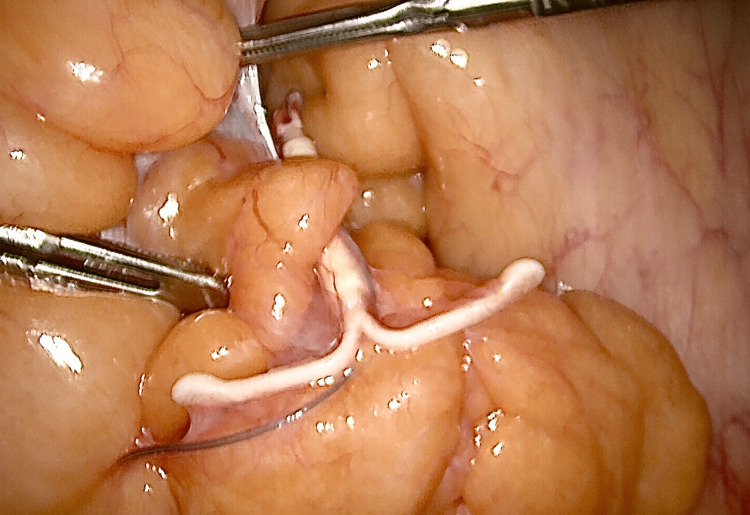
A further view of the intra-abdominal LNG-IUD. LNG-IUD: levonorgestrel-releasing intrauterine device

At laparoscopy, the uterus was found to be retroverted and retroflexed, revealing the cesarean section scar with no evidence of uterine wall damage. The hysteroscopic inspection of the uterine cavity revealed no portion of the coil embedded in the uterine wall. The LNG-IUD was found to be located densely adhered to fatty tissue that was covering the descending colon around the level of the pelvic brim (Figure 2 and Figure 3).

Sharp adhesiolysis with scissors allowed the freeing of the LNG-IUD with no disruption of the bowel walls, and then, it was extracted through a 10-mm port. The device was inspected to verify its integrity. At the end of the procedure, extensive lavage was performed using Hartmann’s solution, and hemostasis was ascertained. A loop cervical diathermy procedure was performed as well to treat CIN 3. The operating time was 105 minutes. The patient was discharged home the following day. Apart from a mild umbilical wound infection treated with oral antibiotics, her recovery was unremarkable. Her husband was booked for a vasectomy operation in the following month.

## Discussion

The first perforation of the uterus by a coil (Grafenberg ring) was reported by Murphy in 1933 [4]. Over the years, perforation of the uterus by IUD has become a rare phenomenon with a reported overall incidence of 1.1-1.4 in 1,000 insertions [5]. In particular, in New Zealand, it was found to be 1.6 in 1,000 insertions of Multiload Cu375 [6] and 0.9 in 1,000 insertions of LNG-IUD [7] when performed mainly by general practitioners.

It has been postulated that uterine perforation may occur at the time of primary insertion or later on caused by gradual damage to the uterine wall. The force required to insert an IUD was found to be 1.5-6.5 N, but the physiological uterine forces have been estimated to be as high as 50 N, enough to produce uterine perforation after the IUD has been placed correctly in the uterus [8]. This would explain a perforation at a later stage.

The patient we present did not have the IUD inserted in the following six months after delivery nor she was breastfeeding at the time of the insertion, which are factors found to increase the risk of uterine perforation by IUD [9-12]. The only possible risk factor in her case was uterine retroversion and retroflexion as it has been reported that 42% of perforations occur in retroverted uteri [13]. Our patient did have a previous cesarean section, but this factor has been found not to increase the risk of uterine perforation [14].

If IUD threads are missing, the first step is requesting a vaginal ultrasound with the caveat that LNG-IUDs such as Mirena (Bayer, Auckland, New Zealand) levonorgestrel 52 mg are harder to identify at ultrasound examination as they only produce an “acoustic shadow,” and they do not present itself as such. As this was the situation in our case, we did request a frontal and sagittal abdominopelvic X-ray that revealed the above IUD in the abdominal cavity. Computed tomography and magnetic resonance imaging have also been suggested for a more precise location of an extrauterine IUD [15], but they are more expensive.

In a report of 701 cases of uterine perforation with an LNG-IUD, 91.5% of the perforations were not diagnosed at the time of the insertion [16], with more than 50% of perforation detected more than a year after the insertion, with the most common symptoms being abdominal pain and the absence of IUD threads or an unexpected pregnancy [6]. The absence of periods, on the other hand, is not a reliable indication that an LNG-IUD is in place, as even when located in the abdomen an LNG-IUD does continue producing levonorgestrel, which has been explained to cause anovulation [17].

There are studies reporting a very low risk of damage to intra-abdominal organs [10,18] in 23 and 75 reported cases of uterine perforation respectively, suggesting a very conservative non-surgical approach in asymptomatic women. Other studies, however, have identified bowel perforation even in asymptomatic patients [13], suggesting the surgical removal of IUD as soon as the diagnosis of uterine perforation is made, especially as they found a positive correlation between time after insertion and the number of adhesions found at surgery. All studies agree, however, that in cases of uterine perforation, copper IUD causes more adhesions than LNG-IUD [13,18], a phenomenon likely due to the toxic effects of copper ions and the ability of copper IUD to cause local inflammation [19]. Therefore, the surgical removal of an abdominal copper coil is likely to be more challenging than in the case of an LNG-IUD.

Operative laparoscopy, as performed in this case, is the preferred method for removing a perforated IUD, and its safety has been advocated widely [13,20].

## Conclusions

Although LNG-IUDs are considered safe, there is a small chance of uterine perforation in about 0.9-1 in 1,000 insertions when performed mainly by general practitioners. It is important to know that more than 90% of perforations are not diagnosed at the time of insertion and more than 50% of perforations are detected more than a year after the insertion. Given a positive correlation between the time of insertion and the development of intra-abdominal adhesions with the possibility of severe damage to intra-abdominal organs, physician awareness is crucial to make an early diagnosis, especially if there is a lack of visualization of the IUD threads, that should prompt the physician to request further investigations such as a vaginal ultrasound and on the suspicion of uterine perforation, laparoscopy should be strongly considered.
